# Outbreaks of mumps genotype G viruses in the Netherlands between October 2019 and March 2020: clusters associated with multiple introductions

**DOI:** 10.1186/s12879-021-06702-7

**Published:** 2021-10-04

**Authors:** Anita A. Shah, Rogier Bodewes, Linda Reijnen, Timo Boelsums, Claudia M. Weller, Ewout B. Fanoy, Irene K. Veldhuijzen

**Affiliations:** 1 Center for Infectious Disease Control, National Institute for Public Health and Environment (RIVM), Bilthoven, The Netherlands; 2 Department of Infectious Disease Control, Public Health Service Rotterdam-Rijnmond (GGD), Rotterdam, The Netherlands; 3 European Programme for Intervention Epidemiology Training (EPIET), European Centre for Disease Prevention and Control (ECDC), Stockholm, Sweden

**Keywords:** MMR, Mumps, Surveillance, Genotype G viruses, The Netherlands

## Abstract

**Background:**

From October 2019–March 2020, several clusters of mumps cases were identified in the Netherlands. Our objective was to describe cluster-associated mumps virus transmission using epidemiological and molecular information in order to help future mumps outbreak investigation and control efforts.

**Methods:**

An epidemiological cluster includes ≥ 2 mumps cases with at least an epidemiological-link to a laboratory-confirmed mumps case. A molecular group includes ≥ 2 mumps cases with identical mumps virus sequences. Cases with symptom onset date between 1 October 2019 and 31 March 2020 reported through the National Notifiable Diseases Surveillance System were included. We described epidemiological and clinical characteristics of mumps cases. Sequence data was obtained from selected regions of mumps virus genomes (2270 nucleotides). Associations between epidemiological and molecular information were investigated.

**Results:**

In total, 102 mumps cases were notified (90% laboratory-confirmed, 10% epidemiologically-linked). 71 out of 102 cases were identified as part of an epidemiological cluster and/or molecular group. Twenty-one (30%) of 71 cases were identified solely from epidemiological information, 25 (35%) solely from molecular surveillance, and 25 (35%) using both. Fourteen epidemiological clusters were identified containing a total of 46 (range: 2–12, median: 3) cases. Complete sequence data was obtained from 50 mumps genotype G viruses. Twelve molecular groups were identified containing 43 (range: 2–13) cases, dispersed geographically and timewise. Combined information grouped seven epidemiological clusters into two distinct molecular groups. The first lasting for 14 weeks, the other for 6. Additionally, one molecular group was detected, linked by geography and time but without an epidemiological-link.

**Conclusions:**

Combined epidemiological and molecular information indicated ongoing mumps virus transmission from multiple introductions for extended time periods. Sequence analysis provided valuable insights into epidemiological clustering. If combined information is available in a timely manner, this would improve outbreak detection, generate further insight into mumps transmission, and guide necessary control measures.

**Supplementary Information:**

The online version contains supplementary material available at 10.1186/s12879-021-06702-7.

## Background

Mumps is an acute infectious disease caused by a paramyxovirus and is usually spread human-to-human by direct contact with respiratory droplets of a person infected with mumps. The incubation period is 16–18 days and the infectious period commences 2 days prior and up to 5 days following the onset of symptoms. Mumps can usually be characterised by parotitis (swelling of the parotid gland) or other salivary gland swelling and the disease is usually mild, however, complications can occur which may include meningitis, orchitis or encephalitis [1]. Molecular surveillance provides a deeper understanding of mumps transmission in near real time by allowing source case identification in clusters and outbreaks, clarification of transmission chains, and detection of genome changes that may influence disease severity or vaccine effectiveness and diagnostics [2–6]. The combination vaccine against measles, mumps, and rubella (MMR) was first introduced in the Netherlands in 1987 for all children aged 14 months and 9 years as part of the Dutch National Immunisation Program [7]. Following the introduction of the MMR vaccination program, the incidence of mumps decreased in the Netherlands, however, several mumps outbreaks have been detected since then. In 2004, an outbreak occurred at an international hotel school [8], and in 2007–2008, an outbreak occurred predominantly in a religious community that had a low vaccination coverage [9]. The largest outbreaks since then have occurred at the end of 2009 to 2012, mainly affecting student populations with a high vaccination coverage [10, 11]. Explanations for these outbreaks could be the possibility of waning immunity in individuals who are appropriately immunised [12]. Other factors may include insufficient effectiveness of the mumps component of the MMR vaccine or increased potential transmission via crowded spaces and social gatherings [12, 13].

From October 2019 to March 2020, several clusters of mumps cases were reported to the National Institute for Public Health and Environment (RIVM) in the Netherlands. The first alert regarding an ongoing mumps cluster was received on 25 October and the identified potential source case was reported to have attended a party held on 4 October while symptomatic. In the following weeks, 11 additional mumps cases were reported as part of this cluster with either epidemiological or molecular sequencing links. Subsequently, additional mumps cases were reported from other close-contact settings. As the number of cases was limited, this offered a unique opportunity to analyse these cases. Our primary objective was to identify and describe clusters of associated mumps virus transmission from multiple exposures by using epidemiological information alone, molecular surveillance alone, and using both together. Our secondary objective was to assess whether mumps cases were occurring due to ongoing transmission or from repeated introductions of genetically distinct mumps virus.

## Methods

### Definitions

In the Netherlands, mumps is a notifiable disease under the Dutch Public Health Act [14]. Mumps cases are reported to the national registration system for notifiable diseases (OSIRIS) by the Municipal Health Service who receives the information from the clinicians and medical microbiology laboratories [15]. The notification criteria for a mumps case includes at least one related symptom (acute onset of painful swelling of the parotid or salivary glands, orchitis or meningitis) and laboratory confirmation of infection or an epidemiological link to a laboratory-confirmed case [11].

An *epidemiological cluster* includes 2 or more cases who met the notification criteria and had an epidemiological link either through exposure to a confirmed mumps case, or has had the same exposure as a mumps confirmed case e.g. attended the same event.

A *molecular group* includes 2 or more mumps cases and in which mumps viruses were detected with identical sequence data that was different from other molecular variants detected in the same time period.

### Epidemiological analysis

We reviewed data on mumps cases reported to OSIRIS with a date of symptom onset between 1 October 2019 and 31 March 2020 in the Netherlands. Following notification of the mumps case, epidemiological investigations were conducted by the Municipal Health Services to gain additional information, such as previous contact with a suspected or confirmed case and travel history. Using available information, we performed descriptive analyses of all cases in terms of demographic characteristics, geographical location, import status, vaccination status, mumps complications and hospitalisation status. R software version 4.0.2 was used for statistical analyses and visualisation of epidemiological data.

### RNA extraction and sequencing

Clinical specimens (oral fluid, throat swab and/or urine) from suspected or laboratory confirmed mumps cases with date of onset between 1 October 2019 and 31 March 2020 were submitted to the RIVM for molecular diagnostics and/or for molecular surveillance [16]. Clinical samples were tested for the presence of mumps virus RNA using real time quantitative PCR as described previously [17]. Samples in which mumps virus RNA was detected, were subject to sequencing of the SH gene and the non-coding regions between the N and P, P and M and M and F genes as described previously [18, 19]. Sanger sequencing was performed at BaseClear (Leiden, the Netherlands).

### Phylogenetic analysis

Obtained sequences were manually checked in Bionumerics version 7.6.3 and a phylogenetic tree was built on concatenated sequences with UPGMA (unweighted pair group method with arithmetic mean) and 1000 bootstrap replicates using Bionumerics version 7.6.3 [20]. In addition, a phylogenetic tree was constructed using IQ-tree software via the webserver (W-IQ-TREE) [20–22] with the maximum likelihood method and the transition model + F (TIM + F) model according to Bayesian information criterion (BIC) based on analysis with ModelFinder [23]. Branch support was calculated using the ultrafast bootstrap approach (UFboot) with 1000 bootstrap alignments [24] and the phylogenetic tree was visualised using FigTree v1.4.4 [25]. Mumps virus MuV/Iowa/6/06 (JX287385) was used as reference strain in the phylogenetic analysis. Time-measured phylogeny was performed using BEAST version 1.10.4 [26]. The nucleotide substitution model used was Tamura Nei 93 with empirical base frequencies according to analysis with ModelFinder [23]. A uncorrelated lognormal relaxed molecular clock was used [27] and a Bayesian SkyGrid Tree prior [28]. The Markov chain Monte Carlo (MCMC) chain was run for 100,000,000 states with a sampling frequency of 1 every 10,000 states, resulting in effective sample sizes of at least 300 for all model parameters according to analysis with Tracer v1.7.1. We used TreeAnnotator v1.10.4 to build an maximum clade credibility (MCC) tree with median node heights and a 10% burn-in. The MCC tree was visualised with FigTree v1.4.4 [25]. Nomenclature is based on date of specimen collection (Additional file 1: Figures S1 and S2).

## Results

### Epidemiological analysis

Between 1 October 2019 and 31 March 2020, 102 mumps cases were notified with a date of onset within this period. Of these, 92 (90%) were laboratory-confirmed, and 10 (10%) were epidemiologically-linked. The median age of all cases was 26 years (range 3–71 years). Of all cases, 57 (56%) were male and 31 (31%) were students (secondary education or higher). For 97 out of 102 (95%) cases, the vaccination status was known. Of those, 58 (60%) cases had received two or more MMR doses, 14 (15%) one dose including 2 cases who were not yet eligible for the second dose, 4 (4%) were vaccinated with number of doses unknown, and 21 (21%) were unvaccinated. Of the 21 unvaccinated cases, the median age was 35 years old (range: 3–71). Two patients, aged 21 and 44 years, and vaccinated with two doses, were hospitalised; both reported orchitis. Among the cases not hospitalised, 5 cases reported orchitis. Nineteen cases (19%) acquired the infection abroad and country of infection was unknown for 7 cases (7%).

**Fig. 1:**
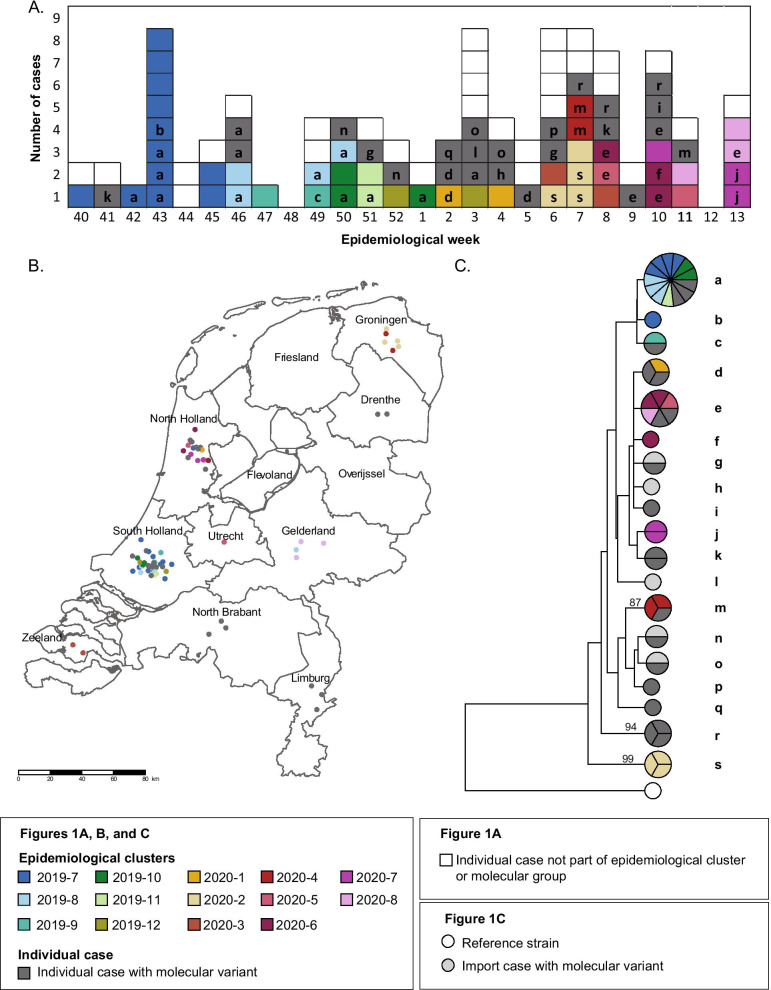
**A** Mumps cases by week of symptom onset between 1 October 2019–31 March 2020, the Netherlands (n = 100 cases)*. **B** Epidemiological cluster (2019-7 to 2020-8) associated cases by province between 1 October 2019–31 March 2020, the Netherlands (n = 71 cases)**. **C** Phylogenetic tree of molecular variants (a–s) of mumps genotype G viruses detected between 1 October 2019–31 March 2020, the Netherlands (n = 50 cases). Bootstrap values > 70 are indicated. *Two individual cases are not shown in **A** as date of onset is not available; one of these cases contains molecular variant c. **For confidentiality reasons, the points in **B** have been jittered

Forty-six of the 102 mumps cases were identified to be part of 14 epidemiological clusters (Table 1, Fig. 1). All 14 epidemiological clusters were identified using epidemiological information alone. The median age among epidemiological cluster-associated cases was 25.5 years (range: 3–71 years). Of all cases, 30 (65%) were male. All of the identified epidemiological clusters contained some close-contact involvement and settings included contact at a party, secondary schools, football match, hotels, and sharing the same household (Table 1).

**Table 1:** Characteristics of epidemiological clusters, the Netherlands, 1 October 2019–31 March 2020 (n = 46 cases)

Epidemiological cluster	Cases	Province of symptom onset	Description of setting	Place of infection of source or co-primary case (if abroad)
2019-7	12	South Holland, North Holland	Party, secondary school	
2019-8	4	South Holland, Gelderland	Football match	
2019-9	2	South Holland	Secondary school	
2019-10	3	South Holland	Hotel, family	
2019-11	2	South Holland	School	
2019-12	2	South Holland	Family	
2020-1	2	North Holland	Friends	Western Europe
2020-2	4	Groningen	Swimming club, family	
2020-3	2	Zeeland	Partners	Western Europe
2020-4	2	Groningen	Holiday abroad	Central Europe
2020-5	2	North Holland, Utrecht	Winter sport trip abroad	Western Europe
2020-6	3	North Holland	Family	
2020-7	3	North Holland	Partners and friend	North America
2020-8	3	Gelderland	Family

Two (2019-10 and 2020-4) of 14 epidemiological clusters had two co-primary cases in each with the same earliest date of symptom onset as the initial source case of their cluster was not identified. The median age of the 16 source cases of each cluster was 22.5 years (range: 5–42 years), 15 cases were vaccinated, and 6 acquired the infection abroad. The place of infection of the source case or co-primary case of the cluster infected abroad was in either Western Europe, Central Europe, or North America (Table 1).

The first identified and largest epidemiological cluster (2019-7) occurred in the provinces South Holland and North Holland, including 12 cases (Table 1, Fig. 1B). The index case had attended a party and was also working at a secondary school. Thereafter, secondary and tertiary cases occurred among attendees of the party and their partners as well as among 4 staff and 1 student at the secondary school. The second epidemiological cluster (2019-8) occurred among attendees of a football match in South Holland and Gelderland. Four additional epidemiological clusters occurred in South Holland (2019-9, 2019-10, 2019-11, and 2019-12). Two of these clusters occurred in school settings, and the other 2 occurred among family members.

For the remaining 8 epidemiological clusters, all contained transmission in close-contact settings among family, friends, or partners (Table 1). Geographically, four of these epidemiological clusters (2020-1, 2020-5, 2020-6, and 2020-7) contained cases reporting onset of symptoms in North Holland. One of the clusters (2020-5) contained cases reporting onset of symptoms in North Holland and Utrecht provinces as they attended a winter sports trip together in another location and returned to their respective provinces of residence. One epidemiological cluster (2020-3) occurred among partners in Zeeland who had travelled abroad. Two epidemiological clusters occurred in Groningen (2020-2 and 2020-4); the first occurring among attendees of a swimming club and their family and the second occurring among two persons travelling on holiday together. The final epidemiological cluster (2020-8) occurred in Gelderland province among family members.

### Molecular surveillance

Using sequence data from the SH gene, a genotype could be obtained from 59 out of 60 mumps cases from which one or more clinical materials were submitted to the RIVM. In 58 cases, a mumps genotype G virus was detected, while in 1 case a mumps genotype C virus was detected. Complete NCRs sequence data could be obtained from 50 mumps genotype G viruses (Genbank Accession numbers MW006669–MW006820). Sequence analysis of these mumps genotype G viruses revealed that at 30 nucleotide positions within the SH + NCRs sequences nucleotide variation was present, which resulted in 19 different molecular variants (a to s) with one or more viruses that had at least one nucleotide difference compared to other mumps viruses (Fig. 1C, Additional file 1: Figure S1). From reviewing the 19 molecular variants, 12 of these contained 2 or more cases. Therefore, 12 molecular groups were identified according to the definition including 43 mumps viruses in total.

### Comparison of results of epidemiological analysis and molecular surveillance

Molecular group a contained the highest number of mumps cases [13]; ten from 4 different epidemiological clusters and 3 individual cases (Fig. 1C). Combined information grouped 4 epidemiological clusters into 1 distinct molecular group. All 13 cases were shown to be dispersed over time with dates of symptom onset occurring over 14 weeks in total, as well as geographically with the majority from South Holland (12/13), and Gelderland provinces (1/13). Three of the 13 cases were not identified as part of an epidemiological cluster.

The second biggest molecular group e contained 6 cases; four from 3 different epidemiological clusters and 2 individual cases (Fig. 1C). Combined information grouped 3 epidemiological clusters into 1 distinct molecular group. Cases had dates of symptom onset over a duration of 6 weeks in total, and cases occurred in 4 different provinces, Gelderland, North and South Holland, and Utrecht.

Four molecular groups (d, m, r, and s) each contained 3 mumps viruses with sequence data. Molecular group r contained cases linked by geography and time but without an epidemiological-link. Molecular group s contained 3 cases from the same epidemiological cluster and cases had dates of symptom onset over a duration of 2 weeks. Molecular group m contained 2 cases from the same epidemiological cluster and 1 individual case. Molecular group d contained 1 case from an epidemiological cluster and 2 individual cases.

The remaining 6 molecular groups each contained mumps viruses detected in 2 cases. Only 1 of these molecular groups contained 2 cases from the same epidemiological cluster (j), while the rest only included individual cases. Overall, 21 of 71 (30%) epidemiologically and/or molecularly-associated cases were identified solely through epidemiological information, 25 (35%) were identified solely from molecular surveillance, and 25 (35%) were identified using both.

### Phylogenetic analysis

In addition to phylogenetic trees prepared using UPGMA (Fig. 1C) and ML method (Additional file 1: Figure S1), mumps virus sequences were analysed with time measured phylogeny (Additional file 1: Figure S2). Bootstrap values or posterior values were low for most branches of the trees. Comparison of the topology of the trees indicated that in each of the trees, molecular groups a, b, and, c and molecular groups m, n, o, p and q belonged to separate branches. Also molecular groups r and s belonged to separate branches in each of the trees, but with different topology. The mean time to Most Recent Common Ancestor (tMRCA) of all mumps viruses included in this study was 27 March 2018 with a 95% high probability distribution (HPD) of 25 May 2015 to 1 October 2019 (Additional file 1: Figure S2).

## Discussion

Our primary objective was to describe mumps cases using epidemiological and molecular sequencing information. Overall, molecular surveillance grouped 7 different epidemiological clusters into 2 distinct molecular group and our findings suggest ongoing mumps virus transmission for extended time periods.

Of interest, sequencing of mumps viruses to understand transmission chains has been the focus of a number of recent studies [2–6]. In the present study, we used a strict definition of a molecular group to compare this with the epidemiological data. However, results of this study and a previous study sequencing the same molecular regions indicated that also within relatively small epidemiological clusters single nucleotide variants occur and it cannot be excluded that two viruses with identical sequences belong to different transmission chains [17]. Therefore, our definition for a molecular group does not necessarily mean that cases of this group belong to the same or different transmission chains. For a similar study with measles virus (with a similar mutation rate [29]), it was concluded that if there are two or more identical nucleotide differences present in the sequences from measles viruses detected in four or more measles cases that occur at the same time, they belong to two different transmission chains. If there are less than two nucleotide difference(s) present between measles viruses detected in two or more cases, it will remain unclear whether they belong to the same or different transmission chains [30]. If we also apply these conclusions to our study, we might conclude that it is unclear whether cases from molecular groups a, b and c belong to one or more transmission chains. On the other hand, molecular group n might belong to a different transmission chain than groups a, b and c. d and o might also belong to two different transmission chains, and the same conclusion might be drawn for cases in molecular groups m, r, s, e and j. The largest molecular group (molecular variant a) contained 13 cases from four different epidemiological clusters. These molecular groups were detected for an extended time period of 14 and 6 weeks, respectively. As molecular clustering together with epidemiological clustering reflects time passed between clusters, this might indicate ongoing transmission of mumps virus of the same molecular variant but we cannot exclude that there were multiple import infections followed by small clusters [4, 6]. Additionally, of interest is the molecular group containing 3 cases with molecular variant r. Cases were closely related by time as the date of symptom onset between cases matched the known incubation period for mumps (16–18 days) [1]. They had a similar geographical location as all were reported from Limburg province, however, no common source or epidemiological link was identified. In this particular instance, molecular sequencing provided valuable information in the absence of a clear epidemiological link and we identified a molecular group which would not have been otherwise determined from epidemiological information [4]. Three molecular groups (molecular variants g, n, and o) each contained 2 individual cases where 1 of the cases was infected abroad. This is particularly interesting as for each group, the 2 cases do not have a similar geographical location and the time between their symptom onset is not reflective of the known incubation period of mumps [1]. These cases might be part of the same transmission chain, or part of different transmission chains with separate introductions. However, both options indicate that there is underreporting of mumps cases.

Bootstrap or posterior values were low for most branches of the phylogenetic trees, most likely as a result of small genetic differences between mumps viruses. Therefore it is not possible to interpret the exact evolutionary development and relationship of mumps viruses analysed in this study. However, comparison of the topologies of the trees might provide some insights into possible transmission chains. In each of the trees, molecular groups a, b, and c and molecular groups m, n, o, p, and q belonged to separate branches. This might indicate ongoing transmission of mumps virus or multiple transmission chains of nearly identical mumps viruses. Mumps viruses in molecular groups r and s belonged to separate branches with high bootstrap support and posterior values in each of the trees, which might suggest that these viruses are part of different transmission chains.

The occurrence of two epidemiological clusters at the same time in Groningen (2020-2 and 2020-4), which is a city with a large (international) student population in the Netherlands, provides an example of the complementary information that sequence data can provide to the epidemiological data. Epidemiological information indicated that infections occurred abroad for one cluster, while the exact source of the other cluster was unknown. Sequence data confirmed that it were indeed two different transmission chains and they could not have been part of one outbreak with low reporting rates.

Use of sequences from known data repositories to compare outbreak strains could help in distinguishing potential international transmission events, which can then be combined with available epidemiological information [4]. Especially if molecular sequencing was available in a timely manner, this would improve early identification of the source case of an outbreak, allow detection of cases imported from abroad, and generate additional clarity on the transmission patterns. This would guide public health interventions, particularly in ongoing large scale outbreaks.

In the present study, concatenated Sanger sequence data was used for molecular surveillance of mumps viruses using the most variable regions of the mumps virus genome. Although results of our study indicated that this provided enough molecular resolution to support epidemiological data, analysis of complete genomes will further increase the molecular resolution [4]. However, the amount of full genomes available on GenBank for mumps viruses is very limited compared to other viruses/pathogens. In many countries, including in the European region, there is currently no or very limited molecular surveillance of mumps viruses. The interest and funding available for molecular surveillance of mumps viruses may change due to the current Covid-19 pandemic, but the molecular surveillance of mumps virus could already greatly be improved if multiple countries start routine molecular surveillance of mumps virus by sequencing the SH gene only, similar to the N450 region for measles virus [31].

Several limitations exist that should be considered when analysing clusters of mumps cases using epidemiological and molecular information at the national level. Molecular sequences available in this study may not be representative for all mumps virus strains circulating during the study period. This can be due to several reasons, including possible underreporting of mumps cases as individuals with symptoms are only notified in the surveillance system if they are laboratory-confirmed or if they have an epidemiological link in the Netherlands. Patients who are asymptomatic and/or who are vaccinated with two or more doses may present with milder symptoms and thus, do not seek health care and there is limited active case finding [2]. Even when patients do visit their general practitioner, laboratory testing may not be conducted for cases and therefore, mumps cases are not reported in the surveillance database. Epidemiological links may be missing from the national surveillance system and hence, it is difficult to determine which cases are part of a specific cluster solely from reviewing the epidemiological information. Thirty-one cases (30%) of the 102 cases had no available epidemiological or molecular information and were dispersed timewise and geographically. If molecular variant information had been available for these cases, this might have provided useful insights in circulating lineages, particularly when there were several epidemiological clusters and molecular variants present, such as between weeks 3 and 13.

## Conclusions

In conclusion, combined epidemiological and molecular information demonstrated ongoing mumps virus transmission for extended time periods. Sequence analysis contributes to surveillance of mumps cases to complement epidemiological clustering. These findings illustrate the importance of combining epidemiological and molecular sequencing information in mumps cluster identification. Cluster information including geographical and temporal distribution of mumps molecular variants, improves outbreak detection and guides implementation of any necessary control measures, especially if available in a timely manner. In addition, it generates further clarity of mumps transmission patterns. This combined information allows earlier outbreak identification and can improve targeted public health recommendations and guidance including broader testing advice and social distancing while being symptomatic.

## Supplementary Information

**Additional file 1: Figure S1.** Phylogenetic analysis of mumps genotype G viruses detected in the Netherlands. **Figure S2.** Time-measured phylogenetic analysis of mumps viruses included in this study.

## Data Availability

The datasets generated during and/or analysed during the current study are not publicly available but are available from the corresponding author on reasonable request. The mumps virus sequences, including metadata, collected for this study are available in GenBank, Accession numbers: MW006669–MW006820.

## Abbreviations

BIC: Bayesian information criterion
HPD: Highest posterior density
MCMC: Markov chain Monte Carlo
MMR: Measles, mumps, rubella vaccine
OSIRIS: National registration system for notifiable diseases
PCR: Polymerase chain reaction
RIVM: National Institute for Public Health and Environment
tMRCA: Time to Most Recent Common Ancestor
UFboot: Ultrafast bootstrap approach
UPGMA: Unweighted pair group method with arithmetic mean
