# Supplementary material for: Outbreaks of mumps genotype G viruses in the Netherlands between October 2019 and March 2020: clusters associated with multiple introductions
Source: BMC Infect Dis. 2021 Oct 4;21:1035. doi: 10.1186/s12879-021-06702-7 (PMC8488918; doi:10.1186/s12879-021-06702-7)
Supplement: Supplementary file 1 — Additional file 1: Figure S1. Phylogenetic analysis of mumps genotype G viruses detected in the Netherlands. Figure S2. Time-measured phylogenetic analysis of mumps viruses included in this study. [file 12879_2021_6702_MOESM1_ESM.docx]

**Outbreaks of mumps genotype G viruses in the Netherlands between October 2019 and March 2020: clusters associated with multiple introductions**

**Authors**

Anita A. Shah^1, 3 *^, Rogier Bodewes^1^, Linda Reijnen^1^, Timo Boelsums^2^, Claudia M. Weller^2^, Ewout B. Fanoy^2^, Irene K. Veldhuijzen^1^

**Affiliations**

1. Center for Infectious Disease Control, National Institute for Public Health and Environment (RIVM), Bilthoven, The Netherlands
2. Department of Infectious Disease Control, Public Health Service Rotterdam-Rijnmond (GGD), Rotterdam, The Netherlands
3. European Programme for Intervention Epidemiology Training (EPIET), European Centre for Disease Prevention and Control (ECDC), Stockholm, Sweden

*Correspondence to [anita.shah@rivm.nl](mailto:anita.shah@rivm.nl)

**Additional file**

**Figure S1. Phylogenetic analysis of mumps genotype G viruses detected in the Netherlands**. SH+NCRs sequence data from 50 mumps viruses detected in the Netherlands in 1 October 2019-31 March 2020 were aligned and phylogenetic analysis was performed using the maximum likelihood method and the TIM+F model. Mumps virus MuV/Iowa.USA/06/6 was used as an outgroup. Epidemiological clusters are indicated next to the virus names and molecular groups are indicated with letters next to branches of the tree. Only bootstrap values >95 are indicated. Nomenclature is based on date of specimen collection.





**Figure S2. Time-measured phylogenetic analysis of mumps viruses included in this study.**

Posterior values (cut-off value 0.5) are indicated at the nodes, while the estimated uncertainty in the date for each node is indicated with 95% PHD credible intervals (light grey bars). Molecular groups are indicated before the virus names, while epidemiological clusters are indicated behind the virus names. Nomenclature is based on date of specimen collection.
